# Improving Carer Experience and Engagement in a Community Rehabilitation Team: A Quality Improvement Project

**DOI:** 10.1192/bjo.2026.11425

**Published:** 2026-06-30

**Authors:** Joe MacDonnell

**Affiliations:** Sheffield Health Partnership University NHS Foundation Trust, Sheffield, United Kingdom

## Abstract

**Aims:**

Working with carers alongside service users improves clinical outcomes, service user experience, and reduces risks of suicide and self-harm. Caring significantly impacts carers' physical and mental health, yet carers often feel unsupported. This project was conducted in a specialist community rehabilitation team in Sheffield supporting 50 service users with significant support needs. The aims of the project were to improve carer engagement and support, and to identify gaps in carer recording.

**Methods:**

A survey based on the Royal College of Psychiatrists standards for community rehabilitation services was distributed electronically to a total of 58 friends, families and carers of current service users. Alongside this an audit of Electronic Patient Records (EPR) was conducted to identify formally recorded carers. This took place in March 2025. The results of this survey and audit were then used to implement changes within the service.

**Results:**

The audit revealed that only 3 out of 50 service users (6%) had formally identified carers recorded, totalling 6 carers. Many more friends and family were recorded without carer status identified.

The response rate of the survey was 24% (14/58). All 14 respondents identified as carers when presented with a definition of a carer as described by Carers UK, suggesting significant under-recording of carers. Carer feedback was generally positive: 93% had emergency contact information, 78% felt well supported by the team, 71% had been offered individual time with team members, and 64% felt involved in care decisions. Areas for development included information around available carers groups, with only 50% being aware these existed, and only 14% of carers being provided with written information about the team.

**Conclusion:**

A bimonthly family, friends, and carers support group was established jointly with the inpatient rehabilitation service. Attendees have given positive feedback regarding this. The written carers pack was reviewed. The team’s view of who constituted a carer was broadened–due to limitations with the EPR carer status wasn’t easily formally recorded, but any family and friends involved in care were considered carers.

This project highlighted gaps in the identification and recording of carers under the community team, despite good levels of carer engagement. The major implemented change was that of a carers group, which was well-received.

